# Antiobesity and hypolipidemic effects of lotus leaf hot water extract with taurine supplementation in rats fed a high fat diet

**DOI:** 10.1186/1423-0127-17-S1-S42

**Published:** 2010-08-24

**Authors:** Huan Du, Jeong-Soon You, Xu Zhao, Ji-Yeon Park, Sung-Hoon Kim, Kyung-Ja Chang

**Affiliations:** 1 Department of Food and Nutrition, Inha University, Incheon, Korea; 2 Department of Chemistry, Konkuk University, Seoul, Korea

## Abstract

**Background:**

Lotus (*Nelumbo nucifera*) leaf has been used to treat obesity. The purpose of this study was to investigate the antiobesity and hypolipidemic effects of lotus leaf hot water extract with taurine supplementation in high fat diet-induced obese rats.

**Methods:**

Four week-old male Sprague-Dawley rats were randomly divided into four groups with 8 rats in each group for a period of 6 weeks (normal diet, N group; high fat diet, HF group; high fat diet + lotus leaf hot water extract, HFL group; high fat diet + lotus leaf hot water extract + taurine, HFLT group). Lotus leaf hot water extract was orally administrated to HFL and HFLT groups and the same amount of distilled water was orally administered (400 mg/kg/day) to N and HF groups. Taurine was supplemented by dissolving in feed water (3% w/v).

**Results:**

The body weight gain and relative weights of epididymal and retroperitoneal adipose tissues were significantly lower in N, HFL and HFLT groups compared to HF group. HFL and HFLT groups showed lower concentrations of total cholesterol, triglyceride and low density lipoprotein cholesterol in serum. HFLT group showed higher the ratio of high density lipoprotein cholesterol/total cholesterol compared to HFL group. HFLT group showed better blood lipid profiles compared to HFL group.

**Conclusions:**

Lotus leaf hot water extract with taurine supplementation showed antiobesity and hypolipidemic effects in high fat diet-induced obese rats, which was more effective than lotus leaf hot water extract alone.

## Background

Recently it has been reported that high fat diets are responsible for high global prevalence of obesity [1,2]. It is well-known that obesity is associated with many chronic diseases in both humans and laboratory animals such as diabetes mellitus, cardiovascular disease, digestive disease, respiratory disease and various cancers [3-5]. Obesity induced by high fat intake is usually accompanied by hyperlipidemia [6] which presents as an abnormally high concentration of lipids in  blood. Generally, this abnormally high concentration of lipids in blood  means elevated blood total cholesterol (TC)  and/or triglyceride (TG) levels [7]. Although hyperlipidemia does not cause any symptoms by itself, these abnormally high blood lipids levels can lead to various cardiovascular diseases (CVD) such as atherosclerosis and coronary heart disease (CHD) [8] which together are one of the most common causes of death in modern society [9].

*Nelumbo nucifera*, known as the sacred lotus, has many medicinal uses in traditional cultures. Previous studies showed that various pharmacologically active substances were separated from different parts of lotus mainly including alkaloids, flavonoids, triterpenoids, polyphenols, steroids and glycosides [10]. Among the different parts, lotus leaf showed a concentration-dependent inhibition of the activities of α-amylase and lipase, and up-regulated lipid metabolism [11]. Taurine (2-aminoethane sulfonic acid) is abundant in the tissues of most mammalians including humans[12,13] and blood taurine concentration was lowered in the obese mice[14]. Taurine supplementation can improve serum lipid profiles in rats [15] and mice[16], and decrease serum TG concentration in overweight or obese humans[17]. In addition, both lotus leaf and taurine have highly safe properties, and can be served as raw materials for functional foods with antiobesity. Therefore, this research was conducted to evaluate antiobesity and hypolipidemic effects of lotus leaf hot water extract with taurine supplementation in rats fed a high fat diet.

## Methods

### Animals and diet

Three-week old male Sprague-Dawley rats were purchased from Hyundai-Bio (Anseong, Korea). All rats were kept at laboratory animal housing at Inha University following the recommendation of the Guide for the Care and Use of Laboratory Animals (Resources 1996) with a constant 12 h light and dark cycle (AM 09:00 ~ PM 09:00), controlled temperature (23 ± 1℃) and humidity (55 ± 10%). Following one week of acclimatization with a pelletized commercial diet, rats were randomly divided into four groups (n = 8) for a period of 6 weeks (normal diet, N group; high fat diet, HF group; high fat diet + lotus leaf hot water extract, HFL group; high fat diet + lotus leaf hot water extract + taurine, HFLT group). Lotus leaf hot water extract was orally administrated to HFL and HFLT groups and the same amount of distilled water was orally administered to N and HF groups. Taurine was supplemented by dissolving in feed water (3% w/v). Food and water intakes were measured everyday and body weight was measured once every two days. The composition of the experimental diet was based on AIN76 [18] as shown in Table 1.

**Table 1:** Composition of experimental diets (g/100g diet)

Ingredients	Experimental diets	
	
	Normal Diet	High Fat Diet
DL-Methione	0.3	0.3
Vitamin mixture	1	1
Mineral mixture	3.5	3.5
Cellulose	5	5
Sucrose	50	40
Corn starch	15	10
Casein	20	20
Bean oil	5	5
Lard	0	15

Carbohydrate (kcal / 100g)	260	200
Protein (kcal / 100g)	85	85
Fat (kcal /100g)	45	180
Total calories (kcal / 100g)	390	465

Vitamin Mixture (g/kg); thiamin hydrochloride 600mg, riboflavin 600mg, pyridoxine hydrochloride 700mg, nicotinic acid 3g, D-calcium pantothenate 1.6g, folic acid 200mg, D-biotin 20mg, cyanocobalamin 1mg retinyl palmitate pre-mix (250,000IU/g) 1.6g, DL-alpha-tocopherol acetate (250IU/g) 20g, cholecalciferol (400,000IU/g) 250mg, menaquinone 5mg sucrose, finely powdered 972.9g, Mineral mixture (g/kg); calcium phosphate dibasic 500g, sodium chloride 74g, potassium citrate monohydrate 220g, potassium sulfate 52g, magnesium oxide 24g, manganous carbonate (43~48%Mn) 3.5g, ferric citrate (16~17%Fe) 6g, zinc carbonate (70% ZnO) 1.6g, cupric carbonate (53~55% Cu) 0.3g, potassium iodate 0.01g, sodium selenite 0.01g, chromium potassium sulfate 0.55g, sucrose finely powdered 118g.

### Preparation of lotus leaf hot water extract

Dried powder of lotus leaf was purchased from Seonwon Temple (Ganghwa-gun, Incheon, Korea). Lotus sample was extracted by water at 90℃ with a solid-liquid ratio of 2.5g/100ml for 2 hours. After vacuum filtration, the extract was introduced into a rotary evaporator (Büchi Laboratoriums Teknik, Switzerland). The concentrated liquid was dried by using a freeze dryer (Ilshin, Korea). The brown powder was obtained with 18.8% extraction yield and it was stored at -20℃ until application.

### Sampling and chemical analysis

After six weeks, the animals were fasted for 12 hours before sacrifice. Blood was collected from the heart and serum was obtained by centrifugation at 3000rpm for 20 minutes. The liver, kidney, spleen, epididymal fat (E-fat) and retroperitoneal fat (R-fat) were weighed. Some of E-fat was removed from the rats for histological photograph. The serums were immediately frozen in liquid nitrogen, and then stored at -70℃ until application (Operon, Korea).

Concentrations of serum TG and total TC were analyzed using automatic analyzer (BPC BioSed srl, Italy). High-density lipoprotein-cholesterol (HDL-C) was obtained from the whole serum with high density lipoprotein precipitation reagent (AM204-1, Asan Pharmaceutical, Korea) after precipitation of low-density lipoprotein and very-low-density lipoprotein for 10 minutes at 3000rpm (Hettich Mikro 200R, Tuttlingen, Germany) [19] and then analyzed for HDL-C using the same method as with TC. Further, the ratio of HDL-C/TC was calculated. Serum low-density lipoprotein cholesterol(LDL-C) value was calculated by using the Friedewald formula [20] as follows:

LDL- C=TC-(HDL-C + TG/5)

Standard serum (lot no. 053801, Asan Pharmaceutical, Korea) was used for calibration before every parameter was analyzed. All of the results were expressed as mg/dl serum.

Histological photograph of adipose tissue was analyzed based on the paraffin method using a light microscope. Fresh tissues were fixed immediately in Bouin’s solution for 6-12 hours and then fixed tissue was washed under running water. After being dehydrated through different grades of alcohol, the tissues were embedded in paraffin block at 60℃. Eight µm sections were cut and mounted on glass slides coated with an egg albumin and then the paraffin was removed with xylen and alcohol. The glass slides were stained with hematoxylin and eosin. After being dehydrated and cleared by alcohol and xylen, the glass slides were mounted in Canada Balsam. Photomicrographs were taken with a Zeiss Axiolab light microscope equipped with a Nikon Microflex HFX microscope camera. The size of epdidymal adipocyte was calculated by Image-Pro Plus 6.0 (Media Cybernetics, Maryland, USA) and the results were expressed as pixels per epdidymal adipocyte.

### Statistical analysis

Data were analyzed for significant difference by one-way analysis of variance followed by Duncan’s multiple range tests at a p<0.05. All analyses were performed using SPSS 17.0 program.

## Results and discussion

### Body weight, diet intake and food efficiency ratio

**Figure 1:**
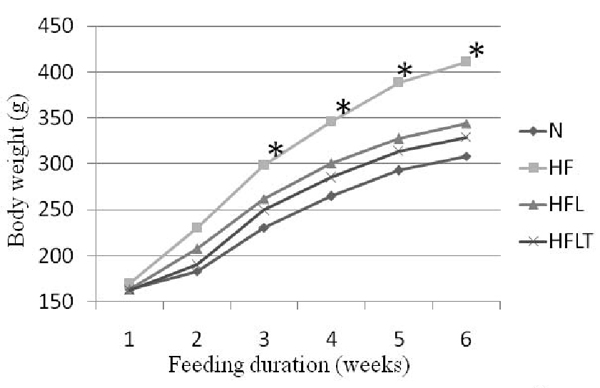
**Effect of lotus leaf hot water extract with taurine on body weight** Average body weights were analyzed by one-way analysis by one way analysis of variance followed by Duncan’s multiple range tests at p<0.05. Superscript stars means that body weight in HF group was significantly higher compared to other groups. N, normal diet group; HF, high fat diet group; HFL, high fat diet + lotus leaf hot water extract group; HFLT, high fat diet + lotus leaf hot water extract + taurine group.

After 3 weeks, body weight of HF group was significantly higher compared to other groups (Figure 1). The food intakes of N and HF groups were higher compared to HFL and HFLT groups. The food efficiency ratio (FER) of N group was significantly lower compared to other groups (Table 2). These results suggest that lotus leaf hot water extract and taurine may prevent an increase of body weight induced by a high fat diet; it seemed that low body weight in HFL and HFLT groups partially due to loss of appetite. In order to understand change of appetite by ingesting lotus leaf hot water extract and taurine, further research will be done.

**Table 2:** Effect of lotus leaf hot water extract with taurine on water intake and FER

Group	Water intake (g/day)			Diet intake (g/day)			FER (%)		
N	29.9	±	1.7^ns^	20.4	±	0.6^a^	24.9	±	0.3^a^
HF	30.8	±	2.7	22.0	±	0.6^a^	29.3	±	1.0^b^
HFL	31.3	±	1.6	17.5	±	0.8^b^	29.6	±	0.5^b^
HFLT	35.4	±	2.0	15.6	±	1.0^b^	30.1	±	0.6^b^

FER: Food efficiency ratio (FER (%) = [total body weight gain (g) / total food intake (g)] × 100%); Values are mean ± SE; Values with different superscripts within the column are significantly different at p<0.05 by Duncan’s multiple range test.

### Organ and adipose tissue weight

The absolute weights of liver, spleen and kidney of HF group were significantly higher compared to other groups. However, there was no significant difference in the relative weight of liver and spleen among groups (additional file 1). Adipose tissue is considered as the biggest storage site for excess energy [21]. The adipose tissue weights (E-fat and R-fat) of HF group were significantly higher compared to other groups. Both absolute and relative retroperitoneal fat weights were significantly decreased around 50% less in HFL and HFLT groups compared to HF group (additional file 2). Therefore, lotus leaf hot water extract alone or with taurine supplementation may inhibit the increase of body fat induced by a high fat diet in rats.

### Serum lipid profiles

Serum lipid profiles are shown in additional file 3. The concentrations of serum TG, TC and LDL-C were significantly lower in HFL and HFLT groups compared to HF group. Lotus leaf hot water extract alone or with taurine supplementation has effects of decreasing the concentration of serum TG, TC and LDL-C, and of increasing the ratio of HDL-C/TC.  These results are in agreement with previous results[11,22,23] and suggest that combined supplementation of lotus leaf hot water extract and taurine showed better blood lipid profiles compared to lotus leaf hot water extract alone.

### Histological photograph

**Figure 2:**
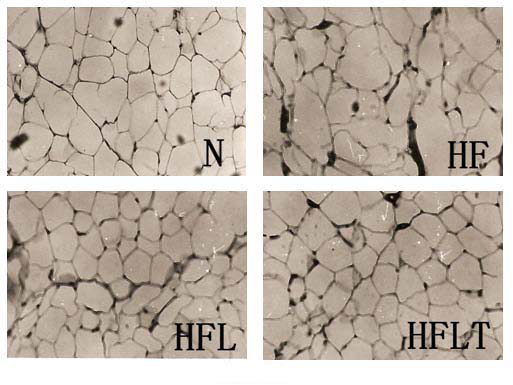
**Effect of lotus leaf hot water extract with taurine on epididymal adipocytes** Histological photograph: Histological photograph of epididymal adipocytes is fed a normal rats’ diet or a high fat rats’ diet with supplementation of lotus leaf hot water extract alone or lotus leaf hot water extract with taurine. Magnification × 200. There was morphological change in HF group compared to other groups. N, normal diet group; HF, high fat diet group; HFL, high fat diet + lotus leaf hot water extract group; HFLT, high fat diet + lotus leaf hot water extract + taurine group.

**Figure 3:**
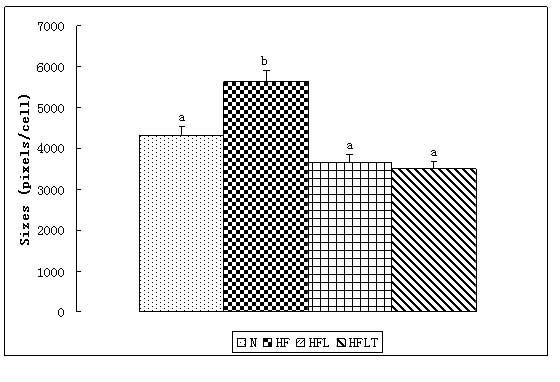
**Effect of lotus leaf hot water extract with taurine on epididymal adipocytes** Sizes of epididymal adipocytes: Effect of lotus leaf hot water extract with taurine supplementation on sizes of epididymal adipocytes in rats fed a normal diet or a high fat diet.  Calculation by stochastic 10 cells by Image-Pro Plus 6.0. Data were presented as mean ± SE. Values with different superscripts are significantly different at p<0.05 by Duncan’s multiple range tests. The size in HF group is significantly bigger compared to other groups. N, normal diet group; HF, high fat diet group; HFL, high fat diet + lotus leaf hot water extract group; HFLT, high fat diet + lotus leaf hot water extract + taurine group.

Excessive growth of adipose tissue results in obesity which includes two growth mechanisms: hyperplastic (cell number increase) and hypertrophic (cell size increase) [24]. The histological appearance of epdidymal adipocyte was irregular in HF group compared to N group. However, this morphological change did not appear in HFL and HFLT groups (Figure 2). The sizes of epdidymal adipocytes were significantly bigger in HF group compared to other groups and the HFL and HFLT groups showed similar adipocyte size to that of N group (Figure 3). These results suggest that lotus leaf hot water extract alone or with taurine supplementation can inhibit lipid accumulation in epididymal adiypocyte tissue.

## Conclusions

In conclusion, our results suggest that supplementation of lotus leaf hot water extract and taurine shows antiobesity and hypolipidemic effects in diet-induced obese rats.

Combined supplementation of lotus leaf hot water extract and taurine showed a better effect on blood lipid profiles as compared to lotus leaf hot water extract alone.

## Abbreviations

FER: Food efficiency ratio (FER (%) = [total body weight gain (g) / total food intake (g)] × 100%); E-fat: epididymal fat; R-fat: retroperitoneal fat; TG: triglyceride; TC: total-cholesterol; HDL-C: high density lipoprotein-cholesterol; LDL-C: low density lipoprotein-cholesterol: TC-(HDL-C+TG/5); HDL-C/TC: ratio of HDL-C/Total Cholesterol

## Competing interests

The authors declare that they have no competing interests.

## Authors' contributions

HD carried out design, execution, statistical analysis, manuscript preparation and total coordination of the study. JSY, XZ, and JYP participated in discussion, data collection, and analysis. SHK provided a method for extracting lotus leaf. KJC guided in the design and execution of the study. All authors read and approved the final manuscript.

## Supplementary Material

Additional file 1 PDF Click here for file

Additional file 2 PDF Click here for file

Additional file 3 PDF Click here for file
