# Supplementary material for: Antiobesity and hypolipidemic effects of lotus leaf hot water extract with taurine supplementation in rats fed a high fat diet
Source: J Biomed Sci. 2010 Aug 24;17(Suppl 1):S42. doi: 10.1186/1423-0127-17-S1-S42 (PMC2994410; doi:10.1186/1423-0127-17-S1-S42)
Supplement: Additional file 1 — PDF [file 1423-0127-17-S1-S42-S1.PDF]

Group	Liver (/100g body weight)	Liver (g)	Kidney (/100g body weight)	Kidney (g)	Spleen (/100g body weight)	Spleen (g)
N	3.26 ± 0.17^{ns}	9.32 ± 0.54^{a}	0.75 ± 0.05^{a}	2.20 ± 0.05^{a}	0.21 ± 0.01^{ns}	0.63 ± 0.04^{ab}
HF	2.90 ± 0.20	11.04 ± 0.83^{b}	0.58 ± 0.01^{b}	2.29 ± 0.09^{b}	0.18 ± 0.01	0.71 ± 0.04^{b}
HFL	3.18 ± 0.14	9.27 ± 0.51^{a}	0.73 ± 0.05^{ab}	2.12 ± 0.06^{ab}	0.18 ± 0.02	0.53 ± 0.06^{a}
HFLT	3.09 ± 0.17	9.29 ± 0.73^{a}	0.75 ± 0.05^{a}	2.23 ± 0.07^{ab}	0.21 ± 0.01	0.62 ± 0.03^{ab}

Values are mean ± SE; Values with different superscripts within the column are significantly different at $p<0.05$ by Duncan's multiple range test.
