# Supplementary material for: Antiobesity and hypolipidemic effects of lotus leaf hot water extract with taurine supplementation in rats fed a high fat diet
Source: J Biomed Sci. 2010 Aug 24;17(Suppl 1):S42. doi: 10.1186/1423-0127-17-S1-S42 (PMC2994410; doi:10.1186/1423-0127-17-S1-S42)
Supplement: Additional file 2 — PDF [file 1423-0127-17-S1-S42-S2.pdf]

Group	E-fat	R-fat	E-fat (/100g)	R-fat (/100g)
N	4.93 ± 0.48[a]	4.53 ± 0.88[a]	1.63 ± 0.10[a]	1.44 ± 0.25[a]
HF	8.91 ± 0.31[b]	11.98 ± 1.47[b]	2.26 ± 0.11[c]	3.00 ± 0.26[b]
HFL	6.21 ± 0.68[a]	5.24 ± 0.84[a]	2.05 ± 0.13[bc]	1.69 ± 0.15[a]
HFLT	6.11 ± 0.89[a]	6.14 ± 1.66[a]	1.92 ± 0.13[abc]	1.78 ± 0.33[a]

/100g: relative adipose weight; E-fat: epididymal fat; R-fat: retroperitoneal fat; Values are mean ± SE; Values with different superscripts within the column are significantly different at $p<0.05$ by Duncan's multiple range test.
