# Supplementary material for: Antiobesity and hypolipidemic effects of lotus leaf hot water extract with taurine supplementation in rats fed a high fat diet
Source: J Biomed Sci. 2010 Aug 24;17(Suppl 1):S42. doi: 10.1186/1423-0127-17-S1-S42 (PMC2994410; doi:10.1186/1423-0127-17-S1-S42)
Supplement: Additional file 3 — PDF [file 1423-0127-17-S1-S42-S3.pdf]

Group	Triglyceride (mg/dl serum)	Cholesterol (mg/dl serum) TC	HDL-C	LDL-C	HDL-C/CHO
N	72.78 ± 12.76[bc]	87.79 ± 8.85[a]	25.72 ± 1.84[b]	47.51 ± 6.38[a]	0.27 ± 0.02[a]
HF	84.06 ± 12.68[c]	112.78 ± 8.34[b]	26.56 ± 2.14[b]	69.41 ± 5.24[b]	0.22 ± 0.01[b]
HFL	58.80 ± 7.16[ab]	90.48 ± 7.13[a]	21.95 ± 1.11[a]	46.72 ± 5.96[a]	0.23 ± 0.01[b]
HFLT	50.61 ± 7.58[a]	88.08 ± 8.46[a]	21.83 ± 0.70[a]	37.21 ± 1.58[a]	0.29 ± 0.01[a]

TC: total-cholesterol; HDL-C: high density lipoprotein-cholesterol; LDL-C: low density lipoprotein-cholesterol: TC-(HDL-C+TG/5)
HDL-C/CHO: ratio of HDL-C/Total Cholesterol; Values are mean ± SE; Values with different superscripts within the column are significantly different at $p<0.05$ by Duncan's multiple range test.
